# Epidemiology of Psychotropic Drug Use in Rio de Janeiro, Brazil: Gaps in Mental Illness Treatments

**DOI:** 10.1371/journal.pone.0062270

**Published:** 2013-05-14

**Authors:** Maria Ines Quintana, Sergio B. Andreoli, Fernanda G. Moreira, Wagner S. Ribeiro, Marcelo M. Feijo, Rodrigo A. Bressan, Evandro S. F. Coutinho, Jair J. Mari

**Affiliations:** 1 Federal University of São Paulo – Paulista Medical School, São Paulo, Brazil; 2 Federal University of São Paulo – Paulista Medical School - Psychiatry Department, São Paulo, Brazil; 3 Santos Catholic University, Santos, Brazil; 4 National School of Public Health – Oswaldo Cruz Foundation, Rio de Janeiro, Brazil; University of Iowa Hospitals & Clinics, United States of America

## Abstract

**Objective:**

Estimate the prevalence of psychotropic drugs use in the city of Rio de Janeiro, Brazil, and establish its relationship with the presence of mental disorders.

**Methods:**

A probabilistic sample of non-institutionalized individuals, from the general population of Rio de Janeiro (n = 1208;turn out:81%), 15 years or older, who were interviewed using the Composite International Diagnostic Interview 2.1 (depression, anxiety-phobia, OCD\PTSD, alcoholism sections), and asked about their psychotropic use during a 12 and one-month period before the interview. Data were collected between June/2007-February/2008.The prevalence was estimated with a confidence interval of 95%. The associations between psychotropics use and mental disorders were analyzed through a logistic regression model (Odds Ration – OR).

**Results:**

The one-month prevalence of psychotropic drug use was 6.55%, 3.19% for men and 9.13% for women. Antidepressants were the most frequently used drug (2.78%), followed by anorectics (1.65%), tranquilizers (1.61%) and mood stabilizers (1.23%). General practitioners issued the highest number of prescriptions (46.3%), followed by psychiatrists (29.3%); 86.6% of the psychotropic drugs used were paid for by the patient himself. Individuals with increased likelihood of using psychotropic drugs were those that had received a psychiatric diagnosis during a one-month period before the study (OR:3.93), females (OR:1.82), separated/divorced (OR:2.23), of increased age (OR:1.03), with higher income (OR:2.96), and family history of mental disorder (OR:2.59); only 16% of the individuals with a current DSM IV diagnosis were using a psychotropic drug; 17% among individuals with a depression-related diagnosis and 8% with Phobic Anxiety Disorders-related diagnosis used psychotropics.

**Conclusion:**

Approximately 84% of individuals displaying some mental disorder did not use psychotropic drugs, which indicates an important gap between demand and access to treatment. A significant failure is evident in the health system for patients with mental disorders; this could be due to health workers' inability to recognize mental disorders among individuals.

## Introduction

The prevalence of psychotropic drug use in the general population varies greatly between countries: 3.5% in England [[1], 6.4% in Chile [2], 7.2% in Canada [3], and 10.6% in Australia [4]. The European Study of the Epidemiology of Mental Disorders/Mental Health Disability: a European Assessment (ESEMed/MHEDEA 2000) [5], performed in six European countries (Belgium, France, Germany, Italy, the Netherlands and Spain), estimated an annual prevalence of 12.3%. The Medical Expenditure Panel Survey (MEPS) in the US reported annual prevalences of 5.9% in 1996, 7% in 2000, and 8.1% in 2001 [6]. According to the National Health and Nutrition Examination Surveys (NHANES) carried out between 1988 and 1994 [7] and 1999 and 2002 [8], the consumption of psychotropic drugs in the American population increased from 6.1% to 11.1%.

An epidemiological study carried out in Rio de Janeiro by Almeida *et. al.* [9] in 1988 in the Governador Island county, estimated a prevalence of 5.2% in psychotropic use. At the beginning of the 90s, Mari *et al.* reported a 12-month prevalence of 10.2% for 4 districts in the city of São Paulo [10], and in 1994 Lima *et al.* [11] found an 12-month prevalence of 9.9% in the South of Brazil, among a population of 328,000 inhabitants.

Although a diagnosis of mental disorder increases the chance of using psychotropic drugs, the existence of a gap between demand and use of these drugs is noteworthy because this difference can reach 80% [5]. Women of older age and with high income are the main users of these drugs [3], [5], [8], [10].

The primary objectives of this study were to estimate the prevalence of psychotropic use in the city of Rio de Janeiro and the factors associated with this use, with emphasis on the presence of mental disorders. The secondary objectives were to identify the main prescribers and clarify how these medications were more frequently obtained.

## Methods

### Settings and study design

The present study was based on data from a larger project titled “Violence and post-traumatic stress disorder in São Paulo and Rio de Janeiro, Brazil.” The detailed study protocol is available in an open-access online journal [12]. A population-based cross-sectional survey was carried out in Rio de Janeiro, Brazil, between June 2007 and February 2008. The sample was representative of the population and included individuals older than 15 years old. The city was initially stratified into seven areas according to homicide rates (index of violence). Subsequently, 30 households per census sector were randomly selected within each stratum. Finally, one resident in each selected household was randomly selected to be interviewed according to Kish's method [13].

### Ethics Statement

This study was approved by the ethical committee of the Federal University of Sao Paulo (process n. 1369/04).

### Measurements

The socio-demographic variables were collected using a questionnaire specifically designed for the study. The variables of interest in this analysis were gender, age, marital status, formal education, income, ethnic group, history of migration, and family history of mental illness; the clinical variable was psychiatric diagnosis one-month prior to the study (CIDI 2.1 DSM IV).

We used the 2.1 version of the Composite International Diagnostic Interview to assess mental disorders (anxiety, depressive disorders, and alcohol misuse/dependence). The CIDI 2.1 is a standardized and fully structured interview that provides psychiatric diagnoses through computerized algorithms according to the Diagnostic and Statistical Manual of the American Psychiatric Association, 4^th^ edition (DSM-IV). The Portuguese version of the CIDI 2.1 has been previously validated and adapted to Brazil's social and cultural context [14]–[16].

Participants were asked the following question: “Have you taken any medication for a nervous breakdown, emotional, psychological, or psychiatric problems, or seizures in the last year?” Individuals who provided a positive answer were subsequently questioned about the use of the drug in the last month, the type of medication, who prescribed it, and where the medication was obtained. Information cards, containing information about generic and trade psychotropic drug names available in the Brazilian market, medical specialties, health professionals, and ways to obtain medications were presented to the respondent. The psychotropic drugs were primarily classified by their pharmacological group, taking into account their main clinical indications.

### Procedures

Face-to-face interviews were performed by a team of lay interviewers provided by the Brazilian Institute of Public Opinion and Statistics (www.ibope.com.br), which is one of the largest Brazilian independent research institutes. The interviewers were trained by researchers from an official CIDI\WHO\UNIFESP Training Center to apply the CIDI. The interviewers were trained by the authors to apply the full set of questionnaires used in the study. To optimize response rates, interviewers visited the selected households up to ten times.

### Statistical analysis

Statistical Package for the Social Sciences 19 (SPSS®) was used. The prevalence of psychotropic use and mental disorders was estimated within confidence intervals of95%. The analysis was adjusted for the complex sample design effect. We focused on psychotropic use and psychiatric diagnosis one month before the study. We used a chi-square test to detect statistically significant associations between the socio-demographic variables and access to medication.

Logistic models were fitted to investigate associations between use of psychotropic drugs, socio-demographic and clinical variables. The first model included variables with p<0.10 from the univariate analysis. The first stage of the modeling process included all variables with p<0.10 from the univariate analysis. In the second stage, variables which p-values became larger than 0.05 were dropped from the model, except for the variable education. Both used the enter method of entry. As shown by Pearce [17] and Reichenheimand & Coutinho [18], the exponential of the logistic regression coefficient can estimate the incidence density ratio (IDR) when the following conditions are met: a) the population is in a steady state during the study period (stationary), i.e., the size of the population is constant across the psychotropic users and non users; b) no selective survival is allowable, i.e., the probability of withdrawal or death from the outcome under this study or other related causes is probably not different across users and non-users; c) the exposure does not seem to influence the survival or recovery probabilities; d) reverse causality is not likely, i.e., the outcome being modeled is not a reciprocal cause on the exposure status; and e) the temporal directionality, from the exposure to the outcome, is sustainable either theoretically or by means of a thorough data collection procedure.

## Results

Interviews were obtained with 1208 respondents (an overall response rate of 81.6%), 56.6% of the participants were women (15–75 years; M:42 years, SD: 16.12), 52.4% living with a partner (52.4%). Fifty-three percent were blacks or mulattos, 69% were natives of Rio de Janeiro, with an average of 9.75 years of school attendance and 27% reported history of mental illness in the family. Sixty-eight percent reported family income of less than US$ 477 per month, 89% were employed at the time of the study (Table 1).

**Table 1:** Distribution of the sample's socio-demographic features (n = 1,208) and prevalence of the use of psychotropic drugs in the month previous to the study (n = 82).

Variables	Total samplen (%)	Prevalence ofpsychotropic drug use% (IC 95%)
Psychotropic consumption		**6.55 (5.05–8.06)**
Gender		
Male	524 (43.5)	3.19 (1.72–4.65)
Female	684 (56.5)	9.13 (6.74–11.52)
Age (years)		
15–19	100 (8.3)	0.77 (0–1.85)
20–29	221(18.3)	1.17 (0–2.59)
30–39	247(20.4)	8.15 (4.39–11.92)
40–49	232(19.2)	5.81(2.58–9.03)
50–59	206(17.1)	12.54 (7.42–17.67)
60–69	135(11.2)	8.06(3.48–12.63)
70–75	67(5.5)	8.55 (1.43–15.67)
Marital status		
married/living with a partner	633 (52.4)	5.96 (3.90–8.01)
widow/er	71 (5.9)	10.42 (2.22–18.62)
separated/divorced	129 (10.7)	13.51 (7.17–19.85)
single	375 (31.0)	4.42 (2.39–6.44)
Employed		
No	87 (11.1)	8.68 (6.00–11.36)
Yes	699 (88.9)	4.91 (3.23–6.59)
Education		
Illiterate	19 (1.6)	2.09 (0–6.23)
4 years	154 (12.7)	7.96 (3.58–12.34)
8 years	284 (23.5)	5.25 (2.31–8.19)
11 years	384 (31.8)	5.43 (2.93–7.92)
>12 years	367 (30.4)	8.32 (5.30–11.33)
Native of		
RJ	831 (68.8)	6.62 (4.80–8.44)
Others	377 (31.2)	6.40 (3.70–9.11)
Religion		
Catholic	656 (54.7)	5.52 (3.66–7.38)
Evangelical	298 (24.8)	6.89 (3.72–10.06)
Spirit	105 (8.8)	7.89 (2.29–13.48)
Atheist	26 (2.2)	no observations
Others	29 (2.4)	12.61 (0–25.60)
Religious	86 (7.2)	10.23 (3.49–16.97)
Race		
White	515 (42.7)	8.20 (5.60–10.80)
Black/mixed	643 (53.4)	4.27 (2.70–5.83)
Others	47 (3.9)	17.12 (4.14–30.10)
Family income		
<159 US$	214 (18.6)	3.61 (0.90–6.32)
160–272 US$	270 (23.4)	5.90 (2.60–9.20)
273–476 US$	301 (26.1)	6.43 (3.39–9.47)
>477 US$	367 (31.9)	8.75 (5.83–11.67)
Family history of mental illness		
No	880 (72.8)	93.45 (91.94–94.95)
Yes	328 (27.2)	6.54 (5.05–8.06)

The only significant difference between non-users and users of psychotropic drugs was average age (41 and 49 years old, respectively; p<0.001, Table 1).

The prevalence of psychotropic drug use was 6.55% (n = 82) and three times higher among women (Table 2). Antidepressants were the most widely used psychotropic drugs (2.78%), especially second-generation psychotropics (2.36%). Women consumed more antidepressants than men (3.65% vs. 0.67%, p<0.001). The second most consumed category of psychotropic drugs was anorectics (1.65%), followed by tranquilizers (1.61%), mood stabilizers (1.24%), and antipsychotics (1.05%). Hypnotics, anticholinergics, barbiturates, medications for treating alcoholism, and others were taken by less than 1% of the studied group. Although point estimates of the various types of drugs were higher in females than males, these differences only showed statistical significance in the case of antidepressants.

**Table 2:** Prevalence of the use of psychotropic drugs, during one month, in the city of Rio de Janeiro, distributed by drug type and gender.

	Male (n = 524)		Female (n = 684)		Total (n = 1208)	
	%	IC95%	%	IC95%	%	IC95%
Psychotropic(general)*	3.19	1.72–4.65	9.14	6.74–11.52	6.55	5.05–8.06
Antidepressive*	1.08	0.19–1.97	4.08	2.47–5.69	2.78	1.79–3.77
1^a^ generation	0.41	0–0.97	0.74	0–1.49	0.59	0.10–1.08
2^a^ generation	0.67	0–0.14	3.65	2.16–5.15	2.36	1.46–3.26
Tranquilizers	1.04	0.18–1.89	2.04	0.76–3.33	1.61	0.79–2.42
Anorectics	0.80	0.10–1.50	2.30	1.01–3.59	1.65	0.86–2.44
Mood stabilizers	0.67	0–1.35	1.67	0.59–2.76	1.24	0.55–1.92
Antipsychotics	0.80	0.01–1.56	1.26	0.42–2.09	1.05	0.47–1.63
typical	0.80	0.02–1.56	1.09	0.32–1.87	0.96	0.41–1.51
atypical	0	0	0.16	0–0.47	0.09	0–0.27
Hypnotics*	0	0	0.40	0–0.97	0.22	0–0.55
Anticholinergics	0.38	0–0.91	0	0	0.17	0–0.40
Barbiturates	0.07	0–0.22	0	0	0.03	0.03–0.10
Alcoholism treatment	0	0	0.16	0–0.47	0.09	0–0.27
Attention Deficit	0		0		0	
Others					1.14	0.56–1.72

* p<0.001.

More than half of the psychotropic users (67.1%; n = 55) used only one psychotropic drug during the month previous to the study, 20.7% (n = 17) used two, and 12.2% (n = 10) used three or more.

The main prescribers were general practitioners (46.3%) followed by psychiatrists (29.3%), cardiologists and neurologists (9.8%), and other doctors (4.9%). The observation that some of the psychotropic drugs were provided by non-medical professionals such as psychologists, dentists, pharmacists, priests, pastors, friends, relatives, and others (11.0%) was a noteworthy finding of this study. Majority of the individuals (86.6%) obtained the drugs with their private resources, 3.7% was given by friends or family and 9.8% through government programs. The classes of psychotropic drugs acquired free of charge through government programs were: antipsychotics (7.7%), antidepressants (8.1%), and tranquilizers (15.4%).

A total of 84% of individuals had at least one one-month mental disorder confirmed by the CIDI and did not use psychotropic drugs. This prevalence was also high in individuals with depressive disorder (72.9%) and phobic anxiety disorder (85.6%) (Table 3).

**Table 3:** Frequencies in the use of psychotropics distributed by type of drug and diagnosis identified in the month previous to the study, in the city of Rio de Janeiro (n = 427).

	Rio de Janeiro			
	n	Benzodiazepines% (n)	Antidepressives% (n)	GAP% (n)
Depression	48	6.3 (3)	16.7 (8)	72.9 (35)
Light depression	20	-	15.0 (3)	70.0 (14)
Moderate depression	12	8.3 (1)	16.7 (2)	75.0 (9)
Severe depression	9	22.2 (2)	22.2 (2)	66.7 (6)
Dysthymia	7	-	14.3 (1)	85.7 (6)
Anxiety-phobias	118	2.5 (3)	7.6 (9)	85.6 (101)
TOC	27	-	7.4 (2)	81.5 (22)
PTSD	28	-	3.6 (1)	71.4 (20)
Any of the above	262	3.4 (9)	6.9 (18)	84.0 (220)

GAP: individuals with positive diagnosis by the CIDI who did not receive psychotropic medication; TOC: Obsessive Compulsive Disorder; PTSD: Post-Traumatic Stress Disorder.

The investigation of the associations between the socio-demographic and clinical variables and psychotropic drug use through multivariate models revealed a positive association with the female gender, increased age, marital status (separated or divorced), psychiatric illness diagnosed in the month previous to the study, family history of mental illness, and belonging to the group with the highest family income range (Table 4).

**Table 4:** Parameter estimates for simultaneous effects of gender, age, education, marital status, race, religion, family income, diagnosed by the CIDI 2.1 (1 month), and family history of mental illness on psychotropic consumption in the month of the study (n = 1208).

	Odds Ratio	Sig.	95% CI	
			Lower	Upper
Sex (female)	1.82	0.03	1.048	3.175
Age	1.03	0.003	1.009	1.048
Education (years)	0.99	0.76	.932	1.052
Marital status				
married		Reference		
single	0.97	0.95	.386	2.446
separated\divorced	2.23	0.01	1.167	4.262
widow/er	1.43	0.26	.768	2.657
Family mental illness	2.59	0.001	1.587	4.228
Family income		.		
<160		Reference		
160–272 US$	1.79	0.21	.716	4.464
273–476 US$	1.95	0.14	.801	4.737
>477 US$	2.96	0.02	1.197	7.297
CIDI diagnosis (positive)	3.93	0.001	2.372	6.512
Constant	0.002	0.000		

The distribution of psychotropic use was similar between the 12-month and one-month in the study. The total consumption prevalence for 12-month was 11.2% (n = 136). The most consumed drugs were antidepressants (4.60%), anorectics, mood stabilizers, tranquillizers, and antipsychotics. There were genders differences in the prevalence of use of anti-depressants and hypnotics (Table 5).

**Table 5:** Prevalence of psychotropic drug use during one year in the city of Rio de Janeiro.

	Male (n = 524)		Female (n = 684)		Total(n = 1208)	
	%	IC95%	%	IC95%	%	IC95%
Psychotropic (general)*	6.56	4.26–8.86	14.72	11.76–17.68	11.18	9.21–13.14
Antidepressive*	1.67	0.56–2.79	6.83	4.70–8.97	4.60	3.28–5.90
1^a^ generation	0.61	0–1.31	1.17	0.19–2.15	0.99	0.30–1.56
2^a^ generation	1.06	0.18–1.93	5.98	4.01–7.95	3.84	2.65–5.03
Anorectics	1.53	0.31–2.76	3.91	2.21–5.60	2.88	1.78–3.98
Mood stabilizers	1.03	0.04–2.02	2.32	1.02–3.61	1.76	0.91–2.61
Tranquilizers	1.04	0.18–1.89	2.30	0.98–3.62	1.75	0.92–2.59
Antipsychotics	0.79	0.02–1.56	1.75	0.70–2.86	1.33	0.65–2.02
typical	0.79	0.02–1.56	1.59	0.59–2.60	1.24	0.58–1.90
atypical	0	0	0.44	0–1.08	0.25	0–0.61
Others	1.58	0.55–2.61	2.20	1.07–3.33	0.84	0.40–1.29
Anticholinergics	0.75	0–1.64	0	0	0.33	0–0.71
Barbiturates	0.07	0–0.22	0.44	0–1.08	0.28	0–0.64
Alcoholism treatment	0.37	0–1.07	0.22	0–0.55	0.28	0–0.65
Hypnotics*	0	0	0.40	0–0.97	0.22	0–0.55
Attention Deficit	0	0	0.57	0–0.17	0.03	0–0.10

* p<0.001.

Over a 12-month period, the odds ratio for diagnosis was 4.21 (IC: 2.79–6.35, p<0,001). The magnitude of the remaining associations showed no significant changes.

## Discussion

This is a report on the associations between the use of psychotropic drugs and presence of various mental disorders in residents in the city of Rio de Janeiro aged 15 and over. The use of psychotropic drugs was 6.55% in a one-month period and the most-used drug class was antidepressants. Variables such as age, female gender, being separated/divorced, having higher income and higher education, mental illness diagnosed in the last year, and family history of mental disorder were independently associated with use of psychotropic drugs in a one-month period of the study. The general practitioners were the main prescribers, the majority of participants in the study paid for the medication themselves, and 84% of the individuals with mental illness in a one-month period, as confirmed by the CIDI 2.1, did not use psychotropic drugs.

The one-month prevalence of psychotropic medication use observed in this study is similar to those found in reports in Chile (6.4%) [2], Spain (6.9%) [19], Canada (7.2%), and USA (5.5%) [7]. However, they were higher than reported in England (3.5%) [1] and lower than reported in Australia (10.6%) [4] as well as southern Brazil (9.9%) [20].The annual consumption prevalence was 11.2%, which was higher than reported in the Netherlands (7.4%) [5] and Germany (5.9%) [5], and lower than reported in France (19.2%) [5], Spain (15.5%) [5], Italy (13.7%) [5], and Belgium (13.2%) [5].

The highest use of psychotropic drugs occurred among women and was associated with increased age and income, which agrees with other epidemiological surveys [3], [5], [7], [21], [22]. Women tend to present higher prevalence of affective disorders, such as anxiety and depression, seek health services more frequently, and show emotional symptoms with greater ease, if compared to men [5], [23], [24]. Alcoholism, which is more frequent in the male population, is inversely associated with use of psychotropic drugs (5). Aparasu *et al.* [25] state that individuals who grow old in good physical health tend to use less psychotropic substances. The negative perception of decreased health status with aging [26], [27], loss of skills, presence of organic chronic diseases leading to an increase in depressive/anxiety episodes, and difficulty sleeping [28] are the factors most commonly associated with increased use of psychotropic drugs with age.

Antidepressants were the most used drugs, primarily among women. The prevalence reported in studies performed since 2000 are similar to the ones reported in the present study and range from 1% in the European study by Ohayon [29], to 6.8% in Chile [2] and Australia [4], and 8.4% in Brazil in 1994 [30]. There has been an increase in use of antidepressants, especially the Selective Serotonin Reuptake Inhibitors (SSRIs) [21], [25], [31]. Zuvekas report a gradual increase in the use of psychotropic drugs from 5.9% in 1996 to 8.1% in 2001, in the US, where the SSRI and other new antidepressants account for 80% of this increase [6].

The consumption prevalence observed in this study for tranquilizers was similar to that described in Australia (1.8%) [4]; however, it was lower than the 3.3% prevalence reported by Galduróz, who evaluated 107 Brazilian cities in 2001 [32] and the 7.6% observed in the Rio Grande do Sul, a Southern state in Brazil, in 1994 [33]. This reduction in the use of tranquilizers might be related to an increase in the number of prescriptions for the use of antidepressants for psychiatric and clinical disorders [25], [26], [34], [35].

The main sources of prescription of psychotropic substances were general practitioners, followed by psychiatrists. In a study by Mari *et al.* in 1989 [21], general practitioners accounted for 46.9% of the issued prescriptions and psychiatrists for only 11.7%. In Brazil, Almeida *et al.* [9] observed that 65.8% of the prescriptions were written by general practitioners in Rio de Janeiro in 1994, whereas in Rio Grande do Sul this percentage was 41% [30]. In England (1) and Norway (2), 80% of the prescriptions for psychotropic drugs were written by general practitioners and only 5.0% by psychiatrists. A possible explanation for the increase in psychotropic drugs prescribed by psychiatrists could be the trend of increasing specialization and increased number of specialized professionals in the market.

We found that only 16% of the individuals with a one-month diagnosis were using psychotropic medication, a situation which is also described by other authors. In Canada, this rate is reported as 19.3% [3], 15.9% in the UK [1], and 32.6% in a multicenter European study [5]. In our sample, only 19% of the subjects diagnosed with moderate to severe depression used antidepressants. Ohayon *et al.* [1] reported in a study in England that 10–40% of individuals with psychiatric diagnosis received a psychotropic drug and only 35% of individuals with depression received some type of treatment with psychotropic drugs; 20% among these used antidepressants. The recognition of the disease by a professional does not guarantee that patients will indeed receive adequate treatment; other studies report that only about 40% will receive appropriate medication and among these, non-adherence may still occur [36], [37].

In the European Study of Epidemiology of Mental Disorders [5], which evaluated diagnosis and medication use during one year in a sample of 21,425 individuals, the proportion of individuals diagnosed with depression who received antidepressants was 21.1%. Of these, 4.6% used only antidepressants and 18.4% used only benzodiazepines. As expected, the frequencies of psychotropic drug use increased with the severity of the disorder.

Drug treatment for anxiety-phobias occurred in 8% of the cases diagnosed by the CIDI 2.1. In this dataset, exclusive use of benzodiazepines was higher than any other drug for some disorders like agoraphobia, social phobia, and panic disorder. Despite the increased access to specialists, in most cases patients did not receive the first-choice medication, which would be antidepressants. This finding is worrisome because inadequate drug-based treatment might cause chronification of symptoms, long-term use of the drug, and consequently drug dependency [38].

Some limitations in this study should be taken into account: a) only diagnoses of depressive disorders, phobic disorders-anxiety, and alcoholism were investigated; b) the diagnoses were carried out through a standardized instrument with all the inherent limitations of the method; c) possible bias in the participants' responses due to difficulty in remembering the drugs used within the last year, or mistaking the commercial names of the drugs in the Brazilian market; d) individuals might have received a prescription and did not follow treatment; e) the diagnosis does not determine the use of medication; disorders with milder/moderate symptoms do not necessarily have to be treated with a psychotropic drug, and these would be false positive cases in the treatment gap; and f) the use of homeopathic drugs or herbal medicines that are often used as tranquilizers was not included in this study.

Comparisons with other studies must be made with caution considering that methodologies could be significantly different. For example, the investigation of psychotropic use is often facilitated by the use of visual material such as photographs, while others studies accepted information provided by family members. Moreover, the investigated period can vary widely (lifetime, 12-month, one-month, or current). Other possible reasons include differences in the historical period in which the data were collected (time frame).

To sum up, a large number of individuals with some mental disorder did not use psychotropic drugs, either because they did not seek help and/or because health professionals did not identify their symptoms as a relevant clinical problem. The results of this study show that even though the number of new professionals in the recent years has increased and social conditions in the country have improved, many individuals with mental disorders are not receiving pharmacological treatment.

Higher income was associated with increased consumption of these drugs. The lack of financial coverage to obtain the medication among the socially underprivileged could explain lower consumption within this group. The identification of the barriers associated with receiving health care, and a better understanding of the stigma related to seeking treatment for mental disorders are imperative to improve the treatment of mental illnesses.
